# Syncope: Advances in Diagnosis and Treatment 2024

**DOI:** 10.1111/jce.16546

**Published:** 2024-12-30

**Authors:** Richard Sutton, Rose Anne Kenny, David G. Benditt

**Affiliations:** ^1^ Department of Cardiology, Hammersmith Hospital Campus, National Heart & Lung Institute Imperial College London UK; ^2^ Trinity College and Director, Faints and Falls Unit St James Hospital Dublin Ireland; ^3^ Arrhythmia Center Mail Code 508 University of Minnesota Minneapolis Minnesota USA

**Keywords:** advances in tilt‐testing methodology, pharmacotherapy in vasovagal syncope, syncope, vasovagal syncope pathophysiology

## Abstract

**Aim:**

In light of many recent advances in the field of vasovagal syncope, a selective review has been undertaken of these developments.

**Methods:**

Recent publications on the following topics were reviewed; understanding of vasovagal syncope pathophysiology, tilt‐testing methodology and interpretation, drug, ablation and pacemaker therapy.

**Results and Conclusions:**

The vasovagal syncope field is very active in researching its pathophysiology, using it to gain better understanding of the process and applying this knowledge to therapy.

## Graphical abstract

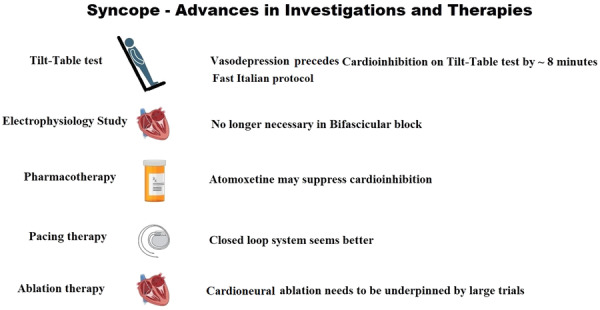

## 1 Introduction

**Figure 1:**
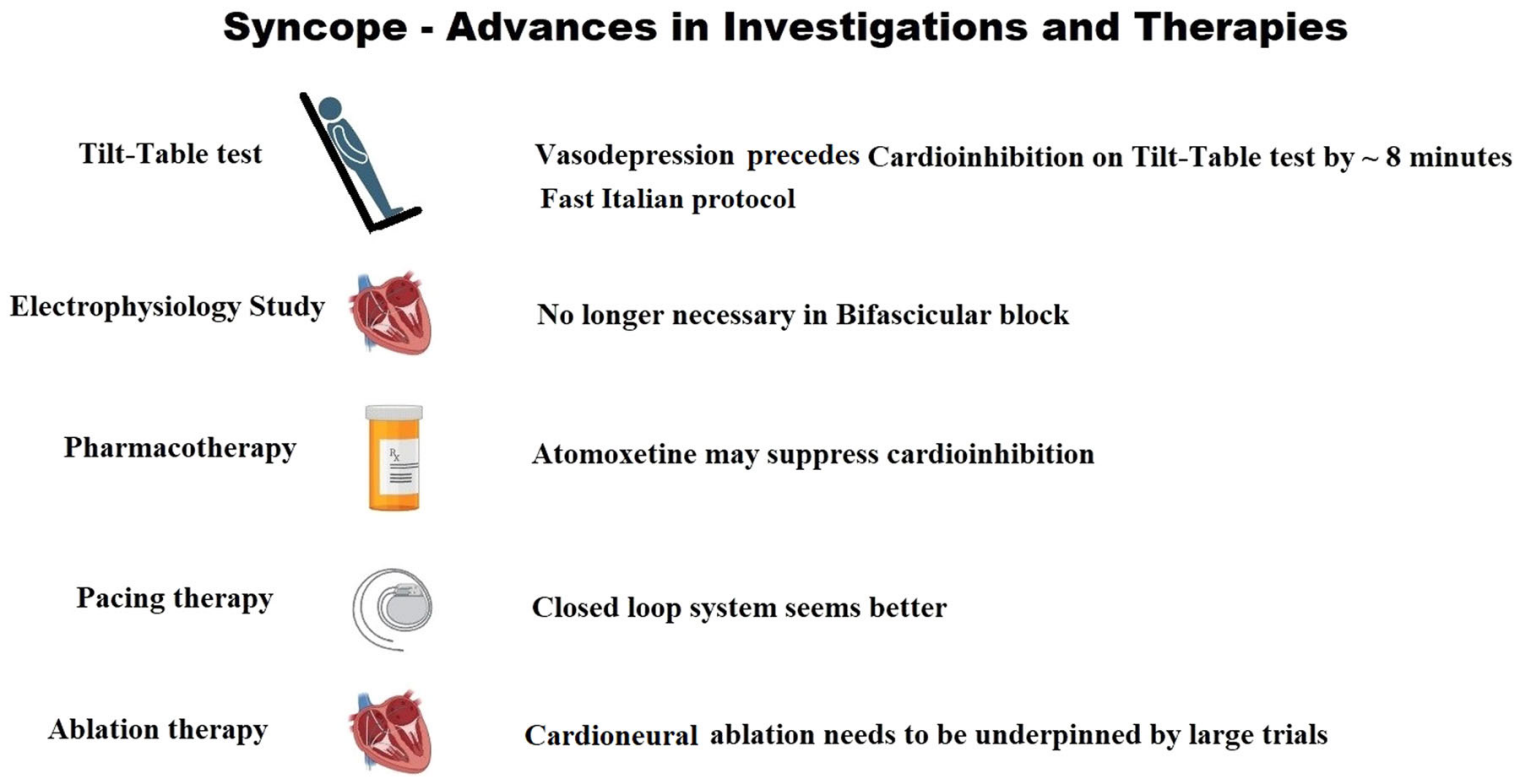
Central illustration. Vasovagal syncope advances in therapy.

Full comprehension of the recent advances in vasovagal syncope (VVS) pathophysiology requires familiarity with the guidelines on syncope management issued by the Heart Rhythm Society (HRS/ACC/AHA) in 2017 [1] and the European Society of Cardiology (ESC) in 2018 [2]. The advances perceived by the authors to be some of the most important are covered under the following five section headings: Better understanding of the evolution of VVS; Important changes in tilt‐testing methodology and interpretation, value of electrophysiological studies (EPS) in prediction of syncope; Drug therapy of VVS; Invasive treatment of VVS (Figure 1 Central Illustration).

## 2 Better Understanding of the Evolution of VVS

The work of van Dijk and his group in Leiden, Netherlands has shown much more clearly the evolution of a typical VVS as induced during tilt‐testing [3]. These researchers have carefully analyzed their series of 163 patients who underwent Tilt‐testing performed with beat‐to‐beat arterial pressure, video‐linked to physiological data to detect head‐flop as onset of transient complete loss of consciousness (TLOC) [4], electrocardiographic and electroencephalographic monitoring. Systemic vascular resistance and stroke volume were derived from the plethysmographic arterial signal by well‐established methods.

Their findings were very clear. Vasodepression (VD) exists in all patients with VVS, furthermore, it begins at approximately 8 min of tilt before Cardioinhibition (CIn) occurs. CIn is almost always present also in VVS providing that the patient is taken to full syncope. The onset of CIn was defined as the point at which the heart rate starts to fall. CIn takes about 1 min to evolve before the test is discontinued by onset of TLOC. Also, during the VD phase the total peripheral resistance (TPR) fell only moderately. During the vasodepressive phase HR rises about 16 bpm. All these changes were highly significant (*p* < 0.001). The authors concluded that the fall in stroke volume was due to venous pooling probably in the splanchnic region, the TPR fall could be attributed to reduced sympathetic drive. CIn only occurred late in VVS evolution, but it contributed importantly to blood pressure (BP) fall and ultimate TLOC. It should be noted that the van Dijk patients were younger than many in other tilt studies.

Attention was subsequently turned, in the same group of patients, to the balance of VD and CIn related to age [5]. The authors concluded that a shift toward less cardioinhibition and more VD with increased age probably reflects physiological changes in the cardiac autonomic nervous system. They also postulated that weakening of cardioinhibition with age may detract from efficacy of pacing in older patients with VVS implying that CIn‐VD balance should influence pacing decisions in this group of patients. Further, van Dijk and colleagues also recognized that asystole in VVS may occur after TLOC rendering it unlikely to be its cause [6]. Thus, selection of pacing therapy in VVS now has these two additional aspects stemming from this center, accessed by thoroughly monitored tilt testing, for consideration of pacing older cardioinhibitory VVS patients.

The finding of age‐related diminution of CIn has been strongly supported by a three‐center study in > 5000 patients [7]. However, some doubt about the strength of validity of this age change has been subsequently raised by the Naples, Italy group [8].

## 3 Developments in Tilt‐Testing

The major recent development in tilt‐testing is really a recapitulation.

Tilt‐testing came to clinical practice in 1986 by the serendipitous discovery that tilt could reveal the mechanism of syncope in older highly symptomatic patients. The revealed mechanism was and is reflex that is, vasovagal.

All the patients in this study were taken to full syncope—complete loss of consciousness [9].

There has since been a tendency to avoid inducing complete loss of consciousness. Perhaps, a feeling of being kind to the patient? This softening of the method is not a service to the patient as it may lead to a wrong diagnosis or reduced interpretability as emphasized in case of possible pacemaker selection as therapy in older VVS patients [6, 10]—see invasive therapy section. With work of the Russo and colleagues we return to the original method [8, 9].

Another valuable modification of the Italian tilt‐testing protocol [11], believed to be the most widely used protocol, also comes from Russo et al. [12] with the shortening of the passive phase of the protocol, labeled the “Fast Italian Protocol,” without prejudicing the results, thus yielding greater laboratory efficiency [13]. The new protocol demands 5 min. supine pretest, 10 min. Head‐up at 70°, administration of 300–400 mcg sublingually and continue head‐up for a further 10 min.

Syncope, even reflex syncope can present diagnostic problems. An important recent advance is the use of ambulatory blood pressure monitoring (ABPM) to support a reflex syncope diagnosis and to understand more of the symptoms and background of these patients [14, 15]; Rivasi et al. [14] have shown that striking asymptomatic BP falls to < 90 mmHg systolic occur frequently in VVS patients. One fall per day of this magnitude has OR 6.2 (*p* < 0.001) with a high specificity but low sensitivity. Such falls are only infrequently seen in controls and, thus, may offer confirmation of the VVS diagnosis in less clear cases.

Emerging practice in the diagnosis of syncope now includes ABPM and Active Stand in addition to carotid sinus massage (vide infra) [16]. Active Stand added to the investigation protocol combined with beat‐to‐beat BP monitoring allows diagnosis of Initial/immediate orthostatic hypotension (iOH) which may be missed on tilt‐testing as it occurs within 45 s of adoption of the standing position. Active Stand may also be used to identify classic orthostatic hypotension (cOH) but not for delayed OH (dOH) which may occur later than the period for which active stand can be maintained. Now, ABPM added to assessment of such patients as those of Torabi et al. [16] will increase the diagnostic potential.

Implantable loop recorders (ILR) are of great value, but they are not always needed. The debate on ILR versus Tilt in syncope assessment has continued over many years [16, 17, 18]. It may be anticipated that the debate will further continue. If choices are subject to financial limitations a detailed workup as described in the work of Torabi et al. [16] is likely to be the less expensive approach.

Carotid sinus massage should be performed in patients > 40 years if there is no contraindication such as recent TIA or stroke. It is recommended that this be done at tilt‐testing usually before the tilt itself [11] to perform it safely, supine and erect, with beat‐to‐beat BP monitoring readily available in the tilt laboratory [19].

In summary of advances in diagnosis and understanding of VVS, when seeking a syncope diagnosis on tilt‐testing, full syncope must be induced. On the other hand, tilts undertaken for patient instruction to recognize prodromes, for example, do not need syncope induction. Tilt‐tests now can be shorter without adversely prejudicing results. Twenty‐four‐hour ambulatory BP recordings show asymptomatic hypotensive episodes in vasovagal patients which increases diagnostic capability. Active stand extends diagnosis to iOH. Carotid sinus massage should be performed on all patients over the age of 40 years.

### 3.1 Value of EPS in Prediction of Syncope

EPS carry restricted recommendations in Guidelines [1, 2] being limited to assessment of patients with bi‐ or trifascicular atrioventricular block (AVB) and when ventricular tachycardia or other potentially ablatable arrhythmia is suspected.

Recently, the vexed question of what to do in syncope presenting with evidence of ventricular conduction tissue disease has been reassessed by Sheldon and co‐workers who, prompted by their own experience, have shown, in a review, that in syncope with bifascicular block EPS has an unacceptable negative predictive value for syncope recurrence [20]. Subsequently, their pragmatic trial of pacing versus ILR included 57 patients, with left bundle branch block or right bundle branch block with left fascicular block [21] (anterior 33, posterior 3) randomized to pacing and 58 to ILR with a follow‐up of 33 months during which recurrent syncope was equally common in the two groups. Adverse outcomes were statistically significantly less in the paced group which prompted the authors to recommend empiric pacing as the best option in this group of patients. These studies may have finally laid this debate to rest. The next Guidelines may be expected to withdraw advice for EPS in syncope with bi‐ or trifascicular block and to make the positive recommendation of empiric pacing although debate may still continue.

### 3.2 Drug Therapy of VVS

The first drug to consider is Midodrine which is not new in therapy of VVS, but it has had little scientific evidence to back its widespread clinical use. The POST 4 trial (Prevention of Syncope Trial) has been published to offer such evidence [22]. POST 4 was a randomized double‐blind placebo‐controlled trial (RCT) of midodrine in 133 VVS patients in 25 centers in Canada, the United States, Mexico, and the United Kingdom.

Patients were assigned 1:1 to placebo/midodrine; they had had a median of six syncopes in the previous year. The primary outcome was one or more recurrence of VVS in 12 months follow‐up. Those who took midodrine (2.5 twice daily to 10 mg three times daily) sustained one or more VVS in 28/66 compared with 41/67 indicating a relative risk on the drug of 0.69, (95% confidence intervals [CI] 0.49–0.97; *p* = 0.035). Number needed to treat (NNT) 5.3 (CI: 2.8–47.6).

The time to first syncope was longer with midodrine hazard ratio (HR) 0.59 (CI: 0.37–0.96); *p* = 0.035; log‐rank *p* = 0.031). Adverse effects were similar between groups. The authors offered limitations that this was a small study size, on young and healthy patients with a relatively short observation period and there was a high proportion of patients from one center. The authors concluded that Midodrine can reduce recurrence of syncope in healthy, younger patients with a high syncope burden. The second drug to consider is Fludrocortisone which has weaker evidence in its favor but is clinically useful despite more side‐effects than Midodrine. The POST‐2 study [23] also stems from Sheldon's group details of which will not be covered here.

Another drug to consider is a new one in the field, Atomoxetine which is a norepinephrine transport inhibitor (NET) currently only used to treat Attention deficit disorder. Atomoxetine and other NETs have shown benefit in small clinical studies of treating VVS patients. POST‐6 from Calgary, Alberta, Canada has shown proof of concept of positive effect of Atomoxetine in tilt studies of VVS patients [24].

Selected patients who had had three VVS in the previous year. On tilt, those receiving two doses of 40 mg Atomoxetine 12 h apart were positive 10/29 and those receiving placebo were positive 19/27, *p* = 0.003: HR 0.49 (95% CI: 0.28–0.86). The beneficial effect appeared to be due to prevention of cardioinhibition.

These results have prompted POST 7 which is a RCT of oral therapy of syncope control in ambulatory VVS patients with a similar symptom profile using Atomoxetine in a dose of 60 mg twice daily versus matching placebo. This trial is currently recruiting. It is tempting to speculate that this drug might have its best effect in the younger VVS patient where Cardioinhibition is most prominent. However, this group of drugs has proved to be challenging in the treating attention deficit syndrome.

Adenosine sensitive syncope (ASS) remains a controversial diagnosis, but most centers see patients with syncope and normal hearts to full investigation, without vasovagal features, and low plasma adenosine. However, few centers are able to access routine plasma levels of adenosine which likely contributes to the controversy surrounding this diagnosis. The method of measurement is now available [25] for when this clinical constellation of findings is recognized.

Empiric treatment with theophylline could be considered. Theophylline is a nonselective adenosine receptor antagonist which makes it eligible for treatment of ASS. A recent trial has assessed this drug in the treatment of ASS where the diagnosis was supported by low plasma levels of adenosine. In 76 ASS patients treated with theophylline 300 mg twice daily (or more according to tolerance) compared with 58 untreated historical controls recurrent syncope occurred in 33% of treated patients and 47% of controls HR 0.52: 95% CI: 0.29–0.93; *p* = 0.029. All were monitored by ILR [26]. The authors concluded that theophylline was effective in preventing recurrences in patients with syncope without prodromes, normal heart, and normal electrocardiogram. It must be noted that side effects may limit long‐term use. It can be anticipated that this group of patients will undergo further studies.

In summary of drug therapy in VVS, Midodrine has at last obtained scientific backing for its clinically experienced efficacy. Atomoxetine may have value in treatment, the RCT is awaited. Its main value may prove to be by blunting Cardioinhibition and delaying or avoiding invasive therapies.

### 3.3 Pacemaker Therapy for VVS

In the last few years, pacing for highly symptomatic vasovagal patients has been well demonstrated by two trials [27, 28] and adopted into ESC Guidelines as a Class 1 indication [10]. The SPAIN trial came first [27]. This was a randomized, double‐blind, controlled trial of VVS patients > 40 years. with 5+ episodes, 2 in last year, positive tilt with LOC and heart rate < 40 bpm for > 10 s or asystole for > 3 s. Randomization was either to DDD‐CLS (closed‐loop‐system) mode in a Biotronik device for 12 months, followed by DDI‐30 bpm for 12 months, (Group A) or vice versa (Group B). Cross‐over was permitted in the event of three syncopes within 1 month. Forty‐six patients completed the protocol, 22 males (47.8%), mean age 56.3 ± 10.6 years, mean previous syncopes 12 [range 9−20]: 72% (95% CI: 47–90) had > 50% reduction of syncopes in DDD‐CLS compared with 28% (95% CI: 9.7–53.5) in DDI‐30 bpm, *p* < 0.017. Four patients (8.7%) had recurrent syncope in DDD‐CLS, 21 patients (45.7%) in DDI, HR 6.7 (95% CI: 2.3–19.8). Kaplan–Meier curves showed time to first syncope recurrence was 29.2 months in DDD‐CLS and 9.3 months in DDI, *p* < 0.016 OR: 0.11 (95% CI: 0.03–0.37, *p* < 0.0001). This was a small study well‐conducted which yielded quite persuasive data.

The SPAIN trial was followed by Bio‐Sync [28] which also employed Biotronik CLS pacemakers in all. This was also a RCT including 127 patients > 40 years (m62). Participants were randomized with 63 to active pacing, and 64 to inactive. Intention to treat analysis was performed. The included patients had sustained an average five lifetime syncopes with > 2 in the last year. Half of them were on hypotensive medications. Patients were finally selected for the study by asystole on tilt in 100% (11% had AVB).

The primary end‐point was time to syncope recurrence. Evaluation of symptomatic events was made by a blind Clinical Events Committee. Interim analysis was performed at 70% of target 62 primary end‐points, allowing the Data, safety, and monitoring Board to terminate the study on the basis of superiority of active pacing. There were five device‐related adverse events, but none had long‐term consequences. The Relative risk reduction at 2 years was 77% and the absolute risk reduction at 2 years was 46%. The NNT was 2.2. The study design did not allow assessment of the relative contribution of the CLS algorithm over conventional dual‐chamber pacing. The authors also concluded that tilt‐testing is a useful method to select VVS candidates for cardiac pacing.

Both SPAIN and BioSync trials have used Biotronik devices with CLS for which it is claimed its response is to ventricular contractility detected from the right ventricle (RV). The exact timing of epinephrine rise and fall, which is associated with contractility, in evolving VVS is uncertain but, as its rise is a response to VD, it is expected to occur after RV volume has started to diminish. VD takes about 8 min to develop in the tilt‐test model of VVS until CIn occurs [3]. The available data from one acute tilt study suggests that CLS triggers pacing about 8 min before cardioinhibition [29], further strengthened by a recent report from the same group [30]. This seems to approximate with CLS sensing RV volume more than RV/LV contractility.

The early response of CLS to trigger pacing before cardioinhibition may explain why the Biotronik device seems to prevent recurrent syncope better than Medtronic's Rate Drop Response which only detects cardioinhibition after a predetermined delay but there has been no long‐term comparative study [31].

The BioSync Trial is definitive with its unique methodology including independent blind assessment of patients' symptoms and its relatively large size. As a result, ESC Guidelines on pacing have changed to recommend pacing in the highly symptomatic, > 40 years, medication resistant, cardioinhibitory VVS patient to being Class 1 Level of Evidence A [10].

### 3.4 Ablation Therapy for VVS

This therapy is now known as cardioneuroablation (CNA) which was introduced by Pachon et al. [32]. Initially, the therapy was slow to gain traction, but in the last 5 years, many series have been published with encouraging results in short‐term efficacy and low complication rate. This has led to pressure from active ablators to offer this therapy widely, however, we still lack randomized trials

In 2023, the first small RCT was published [33], comparing CNA with optimal nonpharmacological therapy in 48 patients with 24 in each group. The primary end‐point was time to first syncope recurrence. This occurred in two CNA patients and in 13 controls (*p* < 0.0004). Quality of Life improved in the CNA patients (*p* < 0.0001) but there was no change in controls over 2 years of follow‐up. The trial looks good, but the numbers are too small to justify general application of this therapy to all symptomatic VVS patients. A Consensus paper has just been published by the European Heart Rhythm Association which attempts to reach a consensus on the attributes and potential dangers of CNA [34].

In conclusion for invasive therapy in VVS, pacing has established itself as the therapy of choice in highly symptomatic VVS patients over the age of 40 years with documented CIn. Closed loop detection of onset of VVS may be better than rate drop response. Below the age of 40 years, there are no trials to guide us. Wider application of pacing requires proof. Here appears to be the role for CNA which is yet to establish itself [35]. Large trials are required.

## Conflicts of Interest

David G. Benditt is consultant and equity holder in Medtronic Inc. The remaining authors declare no conflicts of interest.

## Data Availability

The data that support the findings of this study are available on request from the corresponding author. The data are not publicly available due to privacy or ethical restrictions.
